# L-Mesitran Foam: Evaluation of a New Wound Care Product

**DOI:** 10.1155/2022/4833409

**Published:** 2022-04-14

**Authors:** Segametsi Mary-Jane Mthanti, Gloria Pelle, Niels A. J. Cremers

**Affiliations:** ^1^Prime Cure Medical Centre, 1843 Tsubaki Street, Dube, Soweto 1801, Johannesburg, South Africa; ^2^Safarmex, 85 Newton Road, Meadowdale, Germiston 1614, Johannesburg, South Africa; ^3^Maastricht University Medical Centre, Department of Gynecology and Obstetrics, 6202AZ Maastricht, Netherlands; ^4^Triticum Exploitatie BV, Sleperweg 44, 6222NK Maastricht, Netherlands

## Abstract

Chronic wounds are a health problem that has devastating consequences for patients and their quality of life. Often, chronic wounds are stuck in the inflammatory phase or burdened with an infection. Therefore, it is important to find alternative all-round wound care products that have both wound healing and antimicrobial activities. New wound care products are developed constantly, implementing the latest knowledge and advances in wound care. Honey-based wound care formulations and foam dressings are increasingly used in the clinic. L-Mesitran Foam is a novel product in which a foam dressing is precoated with medical-grade honey. Here, we describe our first experiences with L-Mesitran Foam in the clinic. In this case report, a 57-year-old woman with diabetes mellitus type 2 and hypertension presented with a chronic diabetic venous leg ulcer on her leg. Treatments over six months with different treatments, including povidone-iodine, silver dressings, and compression therapy, were ineffective and subsequently switched to L-Mesitran Foam. The dressing choice was based on the wound type and complied with the instructions for use. Wound healing progressed nicely on different aspects and led to complete healing on day 23. No side effects or pain was experienced during treatment. The presented case supports the safety and efficacy of L-Mesitran Foam and serves as a proof of concept.

## 1. Introduction

Wounds that are stagnated for 4 weeks or are present for more than 8 weeks are considered to be chronic [1, 2]. Chronic wounds are a health problem that has devastating consequences for patients and their quality of life [3, 4]. With chronic wounds accounting for approximately 1–4% of the total healthcare expenditure, it has a major economic impact [3, 4]. Moreover, the expectations are that this will further rise because the number of patients likely increases due to longevity and increased incidences of comorbidities such as diabetes and morbidity [2, 4]. For example, the prevalence of diabetes mellitus has increased from 171 million in 2000 to 451 million in 2017 and is predicted to further increase to 693 million in 2045 [5, 6]. Although wounds in most cases eventually heal by themselves, if there are no underlying pathologies, wound healing can often be improved. Patients frequently use go-to products to disinfect wounds and use regular bandages to keep them clean. For example, the use of povidone-iodine is common practice for most patients to keep the wounds free of pathogens, and this product is often the first go-to product for wounds arising in and around people's homes. Povidone-iodine is an antiseptic and used to disinfect wounds; however, what most patients and nurses do not realize is that it does not promote healing [7]. Some people may still think that dry wounds heal faster, but the research of George Winter proved the concept that moist wounds heal faster than wet or dry wounds [8]. Unfortunately, wound care training in hospitals and universities is limited, and therefore, proper training and more publications about wound care regimens may improve doctors' and nurses' competencies [9, 10].

It is important to find alternative all-round wound care products that have both wound healing and antimicrobial activities. New wound care products are developed constantly, implementing the latest knowledge and understandings and advances in wound care. A new product that has very recently been launched is L-Mesitran Foam. L-Mesitran Foam is a foam dressing that on the one side is coated with L-Mesitran Soft gel. L-Mesitran Soft is a medical-grade honey (MGH)-based wound care formulation. Honey has been used for more than four millennia for wound care [11]. L-Mesitran Foam combines the potent antimicrobial and wound healing effects of MGH together with the strong absorbency capacity of the foam dressing. Both MGH and foam dressings are more and more used for wound care as can be seen by the steadily increased number of publications in PubMed over the last two decades. The search term “honey AND wounds” would yield a maximum of 14 papers per year before the year 2000, while there were 34 publications in 2005, 54 in 2010, and 103 in 2020. For the search term “foam AND dressing,” a maximum of 12 papers about this topic were published before 2000, 19 in 2005, 37 in 2010, and 79 in 2020. We will present our experience on the use of L-Mesitran Foam and support it with a clinical case report.

### 1.1. About the L-Mesitran Foam Dressing: A Combination of Foam with L-Mesitran Soft Foam Layer

**Figure 1:**
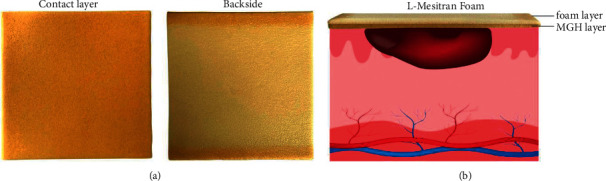
L-Mesitran Foam dressing. (a) Picture of the L-Mesitran Foam dressing showing the two layers: contact layer covered with L-Mesitran Soft and the backside being the foam dressing. The dimensions of the dressing are 10 cm in length, 10 cm in width, and 3 mm thick. (b) Schematic presentation of L-Mesitran Foam covering a wound. The MGH layer creates a moist wound environment and attracts the exudate towards the foam layer which subsequently absorbs it. The foam forms a physical barrier for invading pathogens and protects the wound from mechanical stress.

L-Mesitran Foam is a hydrophilic foam dressing with high absorption and retention characteristics [12, 13]. The sterile foam dressing can be applied as a primary or secondary dressing for wounds with moderate to high exudate and is covered on the one side with L-Mesitran Soft gel (Figure 1(a)). The polyurethane foam forms a cushioning layer that protects the wound from mechanical stress while allowing drainage to pass [12, 13]. Simultaneously, this physical barrier prevents pathogens from entering the wound, while it absorbs excess wound fluid and maintains a moist wound environment [13]. A schematic presentation of these mechanisms is shown in Figure 1(b).

### 1.2. Medical-Grade Honey (MGH)/L-Mesitran Soft Layer

L-Mesitran Foam is coated with L-Mesitran Soft gel and forms good protection to invading pathogens while killing any bacterial loads that are already present [11, 14]. L-Mesitran Soft is known for its antimicrobial and wound healing properties [13, 15, 16] and even exerts prophylactic activity [17, 18]. The antimicrobial effects are orchestrated by its osmotic activity, low pH, the release of hydrogen peroxide, and the presence of several antimicrobial molecules [13, 19, 20]. It has consistently been reported that the supplemented ingredients (such as vitamins C and E) in L-Mesitran Soft enhance the antimicrobial activity of MGH [15, 16, 21, 22]. L-Mesitran Soft has the strongest antimicrobial activity among different honey-based wound care products [13, 16]. The L-Mesitran Soft layer in L-Mesitran Foam creates a moist wound environment which stimulates healing as moist wounds heal faster than dry or wet wounds [8, 23]. Dry wounds result in scabs that disturb healing, while wet wounds lead to maceration and delayed healing [8].

The addition of L-Mesitran Soft to polyurethane foam results in bioactive dressing and establishes regenerative environment [12, 13]. The beneficial antimicrobial and healing properties of L-Mesitran Soft enhance the wound healing process [13, 16]. The introduction of MGH to the wound creates an osmotic effect which further stimulates the release of exudate that is subsequently absorbed and retained by the foam layer [12, 13]. Moreover, during dressing changes, the viscous nature of the MGH layer provides an interface between the wound bed and the foam part and subsequently prevents reopening of the freshly formed granulation tissue [23, 24].

### 1.3. Indications

According to the instructions for use of L-Mesitran Foam, the dressing can be used in the treatment of a wide variety of exuding wounds, such as (diabetic) ulcers, pressure ulcers, superficial and partial thickness burns, fungating wounds, donor sites, chronic wounds, and postoperative wounds.

## 2. Case Presentation

### 2.1. Patient History

**Figure 2:**
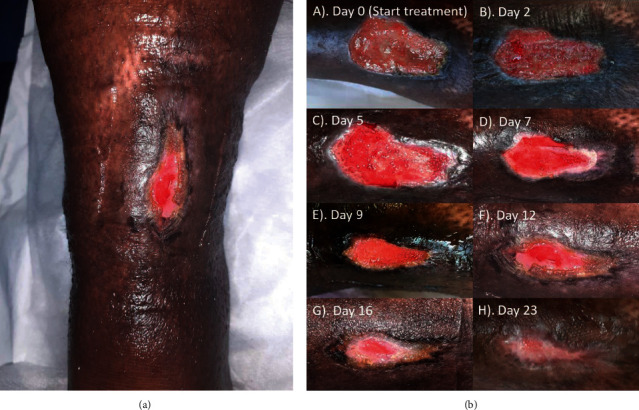
(a) Photo of the wound illustrating the location on the leg. (b) Evaluation of L-Mesitran Foam dressing for the treatment of a chronic diabetic venous leg ulcer. After each dressing change, a picture was taken to monitor the wound progression.

A 57-year-old woman with diabetes mellitus type 2 and hypertension presented to the healthcare clinic with a superficial wound on the anterior aspect of the leg (Figure 2(a)). The diabetes is well-controlled, and the patient compliant with her treatments. The patient reported that the wound was caused by bumping into and being scratched by a steel surface. She has been treating it for six months with different treatments, including compression therapy, povidone-iodine, and silver dressings. Since the wound was becoming bigger and more painful throughout a 6-month period, the patient presented to our clinic providing specialized wound care services after hearing of it.

Originally, the wound was classified as a diabetic ulcer, as the patient is a known diabetic. In our clinic, the wound and patient's conditions were further analyzed, and since the wound was present for six months and there was venous insufficiency, we classified it as chronic diabetic venous leg ulcer. Signs of venous insufficiency included hemosiderin deposit, varicose veins presence, gravity-induced edema, and champagne bottle appearance and were confirmed using Doppler and an ankle-brachial pressure index score of 1.3. The wound was covered with a slimy sloughy looking tissue, suggestive to be a biofilm and being infected, as shown in Figure 2(b), day 0. The dimensions of the wound were about 5.5 cm in length and 3 cm in width.

### 2.2. Methods

The wound was treated with L-Mesitran Foam and compression therapy continued. The dressing choice was based on the wound type. The wound bed showed signs of granulation, low exudate level, presence of low-grade slough, and low odor, with suspicion of infection and the constant shiny appearance suggesting a biofilm. In addition, the wound did not respond to previous treatments and increased in size while not improving the wound edges. The L-Mesitran Foam was introduced as new dressing and was offered to us for testing. Based on its indications for use and our positive experience with the range of other L-Mesitran MGH-based wound care products, we selected the dressing to assess its efficacy in this wound.

The frequency of dressing changes was dependent on the volume of exudate produced. The dressings were initially changed every two to three days for the first two weeks, followed by longer periods up till one week at the end of the treatment. This was to first assess the dressing's ability to absorb and hold the exudate, to monitor the saturation of the dressing, and to enable checking of the wound progression. In the end, dressings were changed once a week because signs of infection disappeared and of the absence of exudate and clear wound healing progression.

The wound size was measured using ImageJ (National Institute of Health, USA, version 1.53k) by setting the scale at 5.5 cm (the original length of the wound) and subsequently performing a “freehand selection” of the wound to determine the area in square cm. Due to the angle of the pictures, there may be a small deviation from the actual area. The relative closure was calculated by dividing the areas of the wounds by the wound area at the start.

## 3. Results

**Figure 3:**
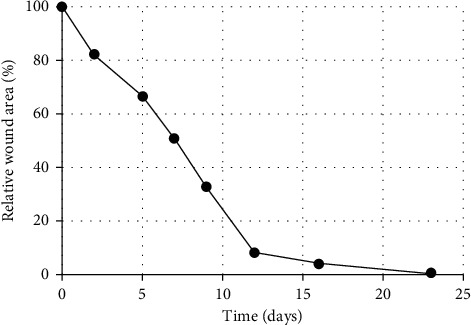
Relative wound closure in time, as quantified using ImageJ in relation to the wound area at the start of L-Mesitran Foam treatment.

Since the wound did not heal for a 6-month period following different treatments, it was decided to treat the wound, which was presumably covered with biofilm and being infected, with L-Mesitran Foam (Figure 2(b), day 0). Because of the signs of venous insufficiency, the dressing was used in conjunction with compression with 40 mmHg. Since there were only local signs of infection, no systemic antibiotics were provided. Diabetic treatment continued as normal, and blood glucose levels were not affected by the treatment with L-Mesitran Foam. Already after one dressing (Figure 2(b), day 2), the sloughy tissue almost completely disappeared, and odor and pain levels strongly decreased. On day five, new granulation tissue was clearly formed, and the first signs of reepithelialization were visible at the edges. Moreover, the tissue looked more vital (Figure 2(b), day 5). After the third dressing (Figure 2(b), day 7), the wound was clean, and reepithelialization was further increased around the edges. The red color supports the tissue to be more vital and neovascularization was evident throughout the new tissue. After 9 days of MGH treatment (Figure 2(b), day 9), reepithelialization and formation of granulation tissue clearly progressed, which continued over the next days (Figure 2(b), days 12 and 16, respectively). The change of color from red into pink shows the wound is further healing and becoming more superficial. After just 23 days, the wound was almost completely closed (Figure 2(b), day 23). The relative wound closure was determined from the pictures and is shown in Figure 3. No side effects or pain were experienced due to the dressing change or use of the L-Mesitran Foam.

## 4. Discussion

L-Mesitran Foam is a hydrophilic foam dressing which is coated on the one side with L-Mesitran Soft. L-Mesitran Soft has antimicrobial activity against a wide range of microorganisms and multiple mechanisms that are responsible for promoting wound healing. In combination with the foam dressing, L-Mesitran promotes the absorption of exudate by its osmotic activity. This osmotic activity attracts wound exudate and lymph fluid from deeper tissue, creating an outflow of fluid [25, 26]. The attracted wound fluid will subsequently be absorbed and retained by the dressing. The L-Mesitran Foam creates the optimal level of moisture and prevents maceration as may happen in wet wounds. L-Mesitran Foam delivers L-Mesitran Soft to the wound bed to foster its wound healing promoting activities. These activities include keeping the wound moist, promoting autolytic debridement, and stimulating granulation tissue formation and reepithelialization [11, 14, 23, 24]. The MGH forms an important nutrient source for the proliferating skin cells [14], but by its stimulatory effect on neovascularization, the transport of nutrients and oxygen from the body will also be improved [25, 26]. The L-Mesitran Foam is user and patient-friendly and can easily be cut to the size of the wound where the soft contact material can serve to protect wound edges and against external mechanical forces by forming a cushioning layer [13]. No pain or discomfort was experienced during the application or change of the dressings. The L-Mesitran Soft layer prevents newly formed granulation tissue to grow into the dressing and allows pain-free removal without reopening of the wound [13, 23, 24]. Even in preterm neonates, the L-Mesitran wound care products are used safely and effectively [11]. Although different L-Mesitran products (Soft, Ointment, Tulle, Hydro, and Net) were already successfully used in a previous study on patients with diabetic venous leg ulcers after previous therapies had failed, the L-Mesitran Foam was not used there [13]. The combination of L-Mesitran Soft with a foam dressing is of added value and can be used to manage a wide variety of exuding wounds. The fast healing of the presented stagnated wound indicates its potential. Despite the convincing results in only one patient and our experience teaching us the wide and efficacious applicability of MGH, the findings should be substantiated in larger patient groups, preferably in double-blinded randomized controlled trials.

## 5. Conclusion

The medical-grade honey of L-Mesitran has strong antimicrobial and wound healing activities. L-Mesitran Foam combines these excellent properties of L-Mesitran Soft with the technology of foam dressings. This fosters the opportunity to absorb and retain wound exudate and to protect the wound from external stressors. The presented case of a chronic diabetic venous leg ulcer serves as a proof of concept of L-Mesitran Foam.

## Data Availability

The data used to support the findings of this study are included within the article.
